# Revisiting the Labial Pit Organ Pathway in the Noctuid Moth, *Helicoverpa armigera*

**DOI:** 10.3389/fphys.2020.00202

**Published:** 2020-03-17

**Authors:** Pramod KC, Xi Chu, Pål Kvello, Xin-Cheng Zhao, Gui-Rong Wang, Bente Gunnveig Berg

**Affiliations:** ^1^Chemosensory Laboratory, Department of Psychology, Norwegian University of Science and Technology (NTNU), Trondheim, Norway; ^2^Department of Teacher Education, Norwegian University of Science and Technology (NTNU), Trondheim, Norway; ^3^Department of Entomology, College of Plant Protection, Henan Agricultural University, Zhengzhou, China; ^4^State Key Laboratory for Biology of Plant Disease and Insect Pests, Institute of Plant Protection, Chinese Academy of Agricultural Sciences, Beijing, China

**Keywords:** LPO sensory pathway, LPOG, antennal lobe, individual sensory neurons, iontophoretic staining

## Abstract

Lepidopteran species detect CO_2_ via a specialized organ located on the peripheral segment of the labial palps, the labial palp pit organ (LPO). Based on tracing of LPO sensory neurons targeting one distinct antennal-lobe glomerulus, Kent and her colleagues described the projections originating from the LPO in the sphinx moth as “*an accessory olfactory pathway in Lepidoptera*” already in the 1980 ties. In spite of similar reports from studies of other lepidopteran species, however, it has been an unresolved issue whether additional termination areas of the labial nerve, such as the gnathal ganglion (GNG) and the ventral nerve cord, are actually output sites of LPO neurons. Since the previous studies have interpreted slightly differently about the projection pattern occurring from the classical mass staining, we performed selective mass staining from the inside of the pit and from the outer surface of the peripheral palp. The results demonstrated that the LPO sensory neurons project exclusively to the LPO glomerulus (LPOG), whereas the non-LPO sensory neurons target the GNG and the ventral nerve cord. Additional iontophoretic staining of individual LPO sensory neurons, performed from the LPO and the LPOG, showed three morphological neuron types: one bilateral targeting the LPOG in both antennal lobes, one unilateral targeting the ipsilateral LPOG only, and one contralateral targeting the LPOG in the other antennal lobe. Finally, to explore putative differences in the projection pattern of neurons housed by two previously reported sensillum types in the pit, i.e., hair-shaped sensilla located distally and club-shaped sensilla located proximally, we performed mass staining from two different levels of the peripheral palp. We found a projection pattern implying stronger innervation of the ipsi- than the contralateral LPOG in both staining experiments.

## Introduction

Many insect species possess the ability to detect even small fluctuations in the atmospheric carbon dioxide (CO_2_) concentration. When it comes to herbivorous insects, this gas is known to serve multiple roles in their interactions with host plants. The sphinx moth, *Manduca sexta*, for instance, is attracted to CO_2_ emitted by freshly opened *Datura wrightii* flowers, which contain large amounts of nectar (Thom et al., 2004; Goyret et al., 2008). Generally, Lepidopteran species sense CO_2_ via a specialized structure called the labial pit organ (LPO; Lee et al., 1985; Kent et al., 1986). It is located on the distal segment of the labial palps where it forms a bottle-shaped cave by being narrow at the tip and wider at the base. Inside there are numerous sensilla containing the sensory neurons detecting CO_2_ (Bogner et al., 1986; Stange, 1992).

Based on staining experiments demonstrating massive innervation in one ventrally located glomerulus in both antennal lobes of several sphinx moths and silk moths, Kent and colleagues described the projections originating from the LPO as “*an accessory olfactory pathway in Lepidoptera”* (1986). The innervated glomerulus was named the LPO glomerulus (LPOG). Notably, all other antennal-lobe glomeruli receive ipsilateral input from olfactory sensory neurons located on the antennae [reviewed by Homberg et al. (1989)]. An unresolved issue, however, is whether sensory axons from the LPO also have projections in central areas other than the antennal lobes. In the classical paper about the “accessory olfactory pathway,” (Kent et al., 1986) found additional innervations in the gnathal ganglion (GNG), and consecutive studies of other moths have reported additional terminal branches in the ventral nerve cord (Lee and Altner, 1986; Zhao et al., 2013; Ma et al., 2017). In all these studies, dye was applied to the truncated part of the peripheral labial-palp segment. Whereas the two last-mentioned reports insisted that all labeled axons were CO_2_-sensitive neurons projecting from the LPO (Zhao et al., 2013; Ma et al., 2017), the original paper suggested that the stained processes in the GNG arose from other sensory neurons on the labial palp (Kent et al., 1986). Thus, even though all previous studies have reported about LPO projections passing bilaterally to the LPOG in each antennal lobe, the total staining pattern including additional axon terminals in other central regions has been interpreted in various ways by the different investigations. Another relevant issue concerns the projection pattern of individual neurons. Although several studies have characterized the LPO sensory neurons, both structurally and functionally (Kent et al., 1986; Stange, 1992, 1997; Stange et al., 1995; Sage, 2002; Guerenstein et al., 2004; Zhao et al., 2013; Ma et al., 2017), we still know little about projection patterns of single axons and how the sampled CO_2_ input is integrated in the central nervous system.

Interestingly, two morphological types of LPO sensilla were recently identified in the heliothine moth, *Helicoverpa armigera* (Zhao et al., 2013). Among the total number of approximately 1200 sensilla, ca. 700 were found to be hair-shaped with a smooth cuticle whereas the remaining 500 were club-shaped with a more grooved surface. The hair-shaped sensilla were found to be located distally in the pit and the club-shaped proximally. Similar sensillum types were reported in the migratory armymoth, *Mythimna separata* (Dong et al., 2014). The distinct projection patterns from the neurons housed inside these sensillum categories are not known, however.

In the present study, we performed staining experiments enabling tracing of the LPO-specific pathway in *H. armigera*. The findings demonstrate that the LPOGs are targeted by axons originating from the pit while the GNG and the ventral nerve cord are innervated by sensory neurons located on the outer surface of the peripheral palp segment. Furthermore, in order to explore putative differences in the projection patterns of neurons housed by the two morphological types of LPO sensilla identified in this species, we carried out anterograde mass staining of sensory axons originating in the pit by applying dye not only to its proximal part but also to the intermediate and distal part. In addition, we performed staining of individual sensory neurons by carrying out iontophoretic labeling from the LPOG as well as the LPO. This gave us the opportunity to trace projection patterns of individual LPO neurons.

## Materials and Methods

### Insects and Preparation

Male *H. armigera* pupae (Lepidoptera; Noctuidae, Heliothinae), purchased from China (Henan Jiyuan Baiyun Industry Co., Ltd), were kept in climate chambers (Refritherm 200 and 6E, Struers-Kebo lab, Albertsund, Denmark, or Binder KBF 720, Tuttlingen, Germany) at 24°C and 70% air humidity on a reversed night-day cycle (14 h light and 10 h dark). The moths were supplied 10% sucrose solution. Experiments were conducted 1–3 days after the emergence. According to Norwegian law of animal welfare there are no restrictions regarding experimental use of Lepidoptera.

### Anterograde Mass Staining of Sensory Neurons Originating From the Third Segment of the Labial Palp

In all experiments, the insect was placed inside a 1 ml plastic pipette (VWR chemicals, France) with the head exposed and then immobilized with dental wax (Kerr Corporation, Romulus, MI, United States). The outer scales of the third labial-palp segment were carefully removed. In order to determine the LPO specific projections, three types of mass staining experiment were performed. To label the projections of the LPO sensory neurons exclusively, the outer wall of the third segment of the labial palp was first sealed with Vaseline. After that, we applied a localized injection by inserting a blunt glass electrode filled with 4% Micro-Ruby (biotinylated dextran-conjugated tetramethyl rhodamine, Molecular Probes) in 0.2 M potassium acetate (KAc) solution into the LPO, with depolarizing current pulses of 7–8 nA at 1 Hz for 2 min. The presence of non-LPO sensory neurons on the labial palp was determined by applying crystals of fluorescent dye on two distinct sites on the outer surface of the third labial-palp segment: (i) a small longitudinal cutting site carefully made on the cuticle of the outer wall and (ii) a transverse cutting site at the very tip of the labial palp.

For investigating the projection pattern of sensory cells housed in the hair-shaped (distal part) and the club-shaped (basal part) sensilla, two kinds of anterograde mass-staining experiments were conducted by cutting at different sites of the labial-palp segment. We first obtained an overview of all the sensory neurons in both sensillar types of the LPO by applying crystals of Micro-Ruby to the transverse cutting plane at its basal part. To label the sensory cells housed in the hair-shaped sensilla exclusively, crystals of the fluorescent dye were applied at the transverse cutting plane made on the intermediate part of the LPO. After staining, the insects were kept for three days in dark to allow anterograde axonal transportation of the dye. A dissecting microscope (Leica M60) equipped with a CCD camera (Leica DMC 4500) was used to determine the location of the transverse cuts made for dye application.

In addition, double-labeling experiments including application of two fluorescent dyes were carried out. One dye was allocated to the intermediate part of the LPO and one to the basal. First, the LPO was sectioned at the intermediate part and then exposed to crystals of Alexa 488. After 24 h at 4^o^C in a humid chamber, the LPO was sectioned at the base and exposed to Micro-Ruby. The insects were then kept for two more days in dark before the brains were dissected.

All brains were dissected out in Ringer’s solution [in mM: 150 NaCl, 3 CaCl_2_, 3 KCl, 25 Sucrose, and 10 N-tris (hydroxymethyl)-methyl-2-amino-ethane sulfonic acid, pH 6.9] before being fixed in a paraformaldehyde solution (4% PFA in 0.1 M phosphate buffer, pH 6.9) for 2 h at room temperature or overnight at 4^o^C. After fixation, the brains were dehydrated in an ascending ethanol series (50, 70, 90, 96, and 2 × 100%; 10 min each), and finally cleared in methyl salicylate (Sigma-Aldrich, Germany). Preparations were then mounted in permount (Chemi-Teknik As, Oslo).

### Iontophoretic Staining of Individual Sensory Neurons

In order to investigate the projection pattern of individual LPO sensory neurons, we first performed iontophoretic staining experiments from the LPO. The insect was immobilized in a plastic pipette as described above. A sharp quartz electrode pulled by a laser-based horizontal puller (P – 2000; Sutter instruments, CA, United States) was used for dye injection. This capillary which contained a fluorescent dye solution (4% Micro-Ruby in 0.2 M potassium acetate solution) was backfilled with 0.2 M KAc and inserted into the LPO via a micromanipulator (Leica microsystems, Wetzlar, Germany). The recording electrode had a resistance of 100–200 MΩ. In addition to anterograde labeling, we performed retrograde labeling by accessing individual sensory neurons projecting to the LPOG. The head cuticle between the eyes was then removed by using a sharp razor-blade knife. The brain was exposed by taking away muscles and tracheas. Likewise, a quartz electrode with dye was inserted into one of the LPOGs. In both types of iontophoretic staining experiments, a chloridized silver wire placed in the eye served as a reference electrode. When the neuron contact was stable, injection of the fluorescent dye was induced via 200 ms pulse depolarizing currents of 8–10 nA for 15 min. After staining, the preparation was kept one to three days at 4^o^C and then dissected, fixed, dehydrated, cleared, and mounted in methyl salicylate.

### Confocal Microscopy

All successfully stained neurons were imaged by using a confocal laser-scanning microscope (LSM 800 Airyscan, LSM 800 GaAsp -Pmt-1, Zeiss, Jena, Germany) equipped with a C- Apochromat 10×/0.45 water objective, Plan-Neofluar 20x/0.5 air objective, and LD LC1 Plan – Apochromat 25×/0.8 imm Korr DIC M 27. The Micro-Ruby (Ex_max_ 555 nm/Em_max_ 580 nm) labeling was excited by the 543-nm line of a HeNe laser, and Alexa 488 (Ex_max_ 495 nm/Em_max_ 519 nm) staining by the 488-nm line of an argon laser. For the samples stained with Micro-Ruby, we used two channels: one for exciting the Micro-Ruby and the other, the 488-nm line of an argon laser, for detecting brain neuropils, made visible via autofluorescence from the sample. We used two detectors: airyscan detecting Micro-Ruby and GaAsP for detecting the autofluorescence. These detectors were used for the double-labeled preparations as well. High-resolution confocal images with 1,024 × 1,024 pixels, at distances of 2 to 6 μm in the z-direction were obtained. The pinhole size was 1 airy unit and the pinhole diameter 36 μm. Images were collected and saved as 8-bit. czi files. In order to quantify the fluorescence intensities in the two LPOGs during dye application to the intermediate and basal segment of the LPO, the 10X water immersion objective with 0.45 NA was used. The image pixel size was set to 0.209 μm and the zoom to 0.8. The images were scanned at 0.55 μs pixel dwelling time. The detector gain was set to 530–630 V, the digital offset to 0, and the digital gain to 1. The axial distance (z thickness) was chosen according to the optimal slicing, i.e., 4.98 μm (having at least 50% overlap of two successive optical sections).

### Image Intensity Processing and Statistical Analysis

All the confocal images were processed in the Zen 2.3 software (Blue edition, Carl Zeiss Microscopy GmbH, Jena, Germany). To estimate the density of sensory projections in the ipsilateral and contralateral LPOG, the fluorescence intensity of each LPOG was quantified by using Image J^1^. Furthermore, in order to compare the staining patterns formed by the two types of sensory neurons housed by hair-shaped and club-shaped sensilla, eight preparations labeled from the base of the pit and six from the intermediate part were included in the analysis. First, we selected the relevant part of the confocal stack, i.e., the images containing the LPOGs. The fluorescence intensities of the LPOGs were then registered through measuring the fluorescence in the glomerular region within the stack. The background fluorescence, i.e., the autofluorescence, was quantified in a corresponding manner from the region between the two LPOGs of the same stack. The LPOG fluorescence intensity per square micrometer was then normalized by subtracting the background fluorescence: mean fluorescence intensity within the LPOG area (μm^2^) minus mean fluorescence intensity within the randomly selected background area (μm^2^). Since the data were not normally distributed, the non-parametric Wilcoxon signed-rank test was performed to analyze the data within subjects. All probabilities given were two-tailed. For analyzes of data linked to the two cutting levels, obtained from different individuals, the Mann-Whitney *U* test was utilized. Effect size (*r*) was calculated by dividing the standardized test statistics by the square root of the sample size (Pallant, 2007). The Statistical package for the social sciences (SPSS), version 25, was used for statistical analysis.

## Results

### LPO-Specific Sensory Neurons Terminate in the LPOGs Exclusively

**FIGURE 1:**
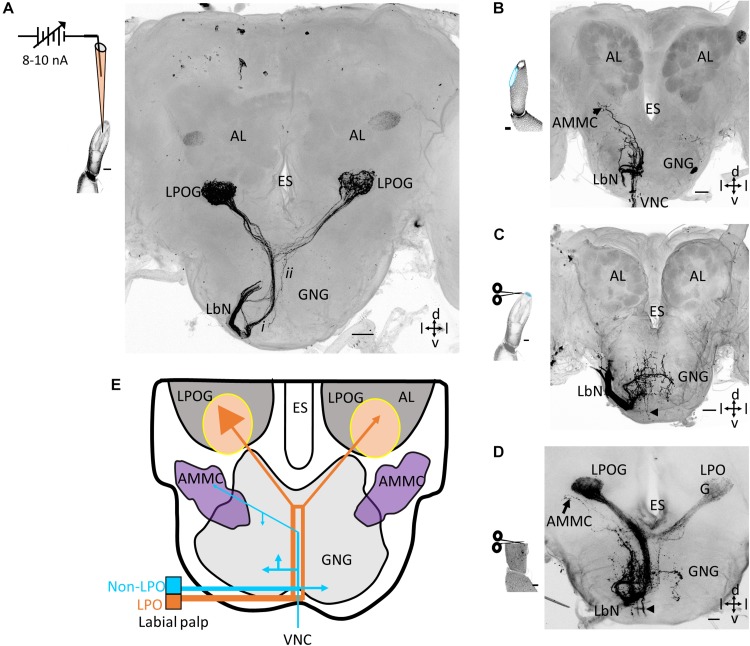
Central projections of neurons in the labial pit organ (LPO) versus non-LPO neurons. **(A)** Confocal image (maximum intensity projection) showing a focused mass staining of sensory neurons housed inside the LPO. As demonstrated, the labeled axons target only the LPOG in both antennal lobes. The labial nerve (LbN) splits into two sub-branches after entering the ipsilateral gnathal ganglion (GNG): one thick main branch (i) and one thin sub-branch (ii). The schematic drawing demonstrates the focused mass staining inside the LPO. The weakly labeled glomerulus located more dorsally in each antennal lobe (AL) is likely visualized due to autofluorescence. **(B)** Projection pattern of sensory neurons from the outer cuticular wall of the labial palp. The axons targeted the GNG and the antennal mechanosensory and motor center (AMMC) on the ipsilateral side. In addition, some fibers projected to the ventral nerve cord (VNC). The schematic drawing indicates the staining site, in *light blue*. **(C)** Projection pattern of sensory neurons originating from the tip of the palp. The axon terminals were bilaterally scattered in the GNG. A few ipsilateral axons projected to the VNC (arrowhead). The schematic drawing indicates the staining site, in *light blue*. **(D)** Projection pattern from a classic mass staining of all sensory neurons including those from the LPO and the outer palp cuticle. The terminal axons targeted the LPOGs in both AL, the GNG, and the VNC (arrowhead). **(E)** Schematic diagram showing the projection pattern of the sensory neurons housed inside and outside the LPO, respectively. The LPO projection pattern in *orange* and the non-LPO in *blue*. The number of endings in the LPOGs represents the intensity of innervations. ES, esophagus; d, dorsal; v, ventral; l, lateral. Scale bars **(A–D)**: 50 μm.

**FIGURE 2:**
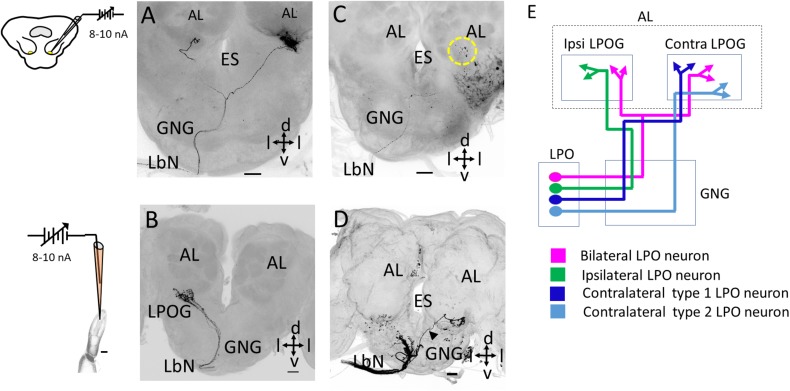
Morphology of individual labial pit organ (LPO) sensory neurons. **(A)** Confocal image (maximum intensity projection) of one bilateral LPO neuron. **(B)** Confocal image (maximum intensity projection) of several co-stained neurons, all projecting to the ipsilateral LPO glomerulus (LPOG). **(C,D)** Confocal images showing projections of two sub-types of contralateral LPO neurons. The neuron in **C**, projects along the main fiber bundle on the ipsilateral side of the gnathal ganglion (GNG) before crossing the midline and targeting the contralateral LPOG. The dotted yellow circle represents the LPOG in the antennal lobe (AL). In **(D)**, one fiber (arrowhead) projecting along the thin axon-bundle on the contralateral side terminates in the contralateral LPOG. The additional fibers in the ipsilateral GNG were assumingly originating from sensilla on the outer palp surface. **(E)** Schematic diagram showing the morphological types of LPO neurons. ES, esophagus; d, dorsal; v, ventral; l, lateral. The neurons in **A,C** were obtained by retrograde staining from the LPOG whereas the neurons in **B,D** were obtained by anterograde staining from LPO. Scale bars **(A–D)** 50 μm.

Iontophoretic staining experiments performed by inserting a blunt glass electrode into the pit showed that neurons originating from sensilla located inside the LPO project exclusively to the antennal lobes. Here they target the ventrally located LPOG. As shown in one of totally five successfully labeled preparations, an axon bundle projecting via the labial nerve can be seen (Figure 1A). After entering the ipsilateral part of the GNG, it passed on dorsally, close to the midline ipsilaterally in the GNG. About 70 μm ventrally of the esophagus, it divided into two branches, each targeting the LPOG in one antennal lobe. In addition to this main axon bundle projecting ipsilaterally in the GNG, another thin sub-bundle crossed the midline about 170 μm ventrally of the esophagus and projected dorsally on the contralateral side (Figure 1A). Confocal images of preparations stained from both sides, showed that this thin sub-branch merged with the main fiber bundle from the other LPO (Supplementary Figure S1). None of the fiber bundles extended terminal branches in the GNG. The four remaining preparations successfully stained by the same technique showed a similar projection pattern including a denser innervation in the ipsilateral LPOG than in the contralateral one (Figure 2B and Supplementary Figure S2).

To determine the projection patterns of sensory neurons located outside the LPO, we first conducted focused mass staining by applying dye to a longitudinal section carefully made on the outer wall of the third labial-palp segment (Figures 1B,E). Five successfully labeled preparations showed stained fibers entering the GNG via the labial nerve, but unlike the projection pattern of sensory neurons originating inside the pit, no projections targeted the LPOGs. As shown in Figure 1B, the main portion of projections from the outer wall terminated in the ipsilateral GNG. In addition to these innervations, a bundle of fibers projected to the ventral nerve cord and a few axons targeted the ipsilateral antennal mechanosensory and motor center (AMMC). The four remaining preparations stained in the same manner showed similar projection patterns (Supplementary Figure S3). In one preparation, a very weak staining can be observed in the LPOGs – possibly due to dye leakage from the outer cuticle (Supplementary Figure S3E). An additional series of mass staining experiments from the palp surface were carried out by applying dye onto the distal cross section of the third segment (*n* = 5; Figure 1C). Again, a bundle of stained axons confined to the labial palp nerve innervated the GNG. However, unlike the targets of axons originating from the longitudinal section of the palp surface, these fibers terminated not only in the ipsilateral GNG but also the contralateral. Besides, there were only a few axons projecting to the ventral nerve cord and no innervation of the AMMC (Figures 1C,E and Supplementary Figure S4). Noticeably, combining the three projection patterns obtained by applying dye into the LPO and to the two different regions of the palp surface, gives a total pattern very similar to that obtained when applying dye to the transverse section of the peripheral labial-palp, at the proximal level (Figure 1D).

### Morphological Characterization of Individual LPO Neurons

We performed iontophoretic staining with a sharp glass electrode both from the LPO and the LPOG, providing morphological identification of individual LPO neurons via anterograde and retrograde labeling, respectively. The assembly of successfully stained neurons comprised three morphological types – all entering the GNG via the labial nerve and then projecting to the LPOG in the antennal lobe. The first type comprised bilateral neurons projecting to the LPOG in both antennal lobes (Figures 2A–E). The second type was unilateral, targeting the LPOG in the ipsilateral antennal lobe exclusively (Figure 2B). The axons of these two neuron types followed the main fiber bundle ipsilaterally in the GNG. The third neuron type had an axon crossing the midline of the GNG and targeting the LPOG in the contralateral antennal lobe only (Figures 2C,D). This contralateral neuron type included two sub-types. One sub-type followed the main axon bundle on the ipsilateral side of the GNG before crossing the midline and terminating in the contralateral LPOG (Figure 2C) whereas the other sub-type targeted this glomerulus via the thin bundle on the contralateral side of the GNG (Figure 2D, *n* = 2). No other areas of the central nervous system were innervated by the individual neurons.

### Projection Patterns of Sensory Neurons Originating From the Intermedial and Basal Part of the Third Labial-Palp Segment

**FIGURE 3:**
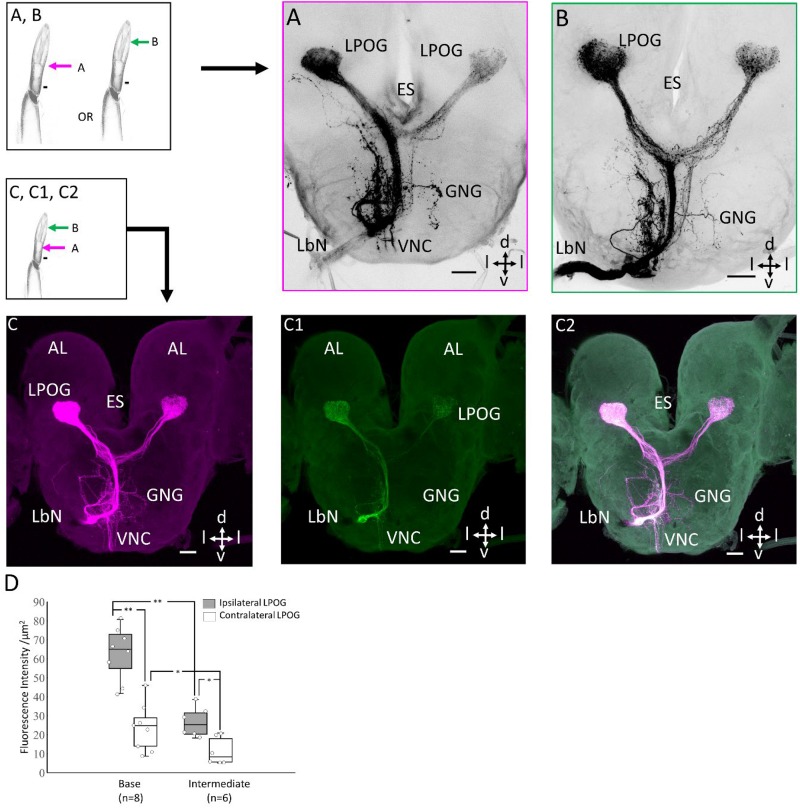
Staining patterns of sensory neurons originating from different segments of the peripheral labial palp. Left upper: Demonstration of application of dye in base or intermediate part of labial palp. Left lower: Schematic diagram showing double-labeling of the labial palp. **(A)** Confocal image of a classic mass staining performed from the transverse cut, at the level of the base of the pit. **(B)** Confocal image of a classic mass staining performed from the transverse cut, at the intermediate level of the pit. **(C–C2)** Double-labeling of the staining experiments performed in **A** and **B** in the same preparation. A difference in fluorescence intensity between the two LPOGs is shown in **C** and **C1**. **(D)** Box plots of quantitative measurements of mean fluorescence intensity in the ipsilateral (*light gray*) and contralateral LPOG (*white*) when the preparation is stained from the base and intermediate part of the labial palp. Wilcoxon signed-rank test *p < 0.05; **p < 0.01. ES, esophagus; GNG, gnathal ganglion; d, dorsal; v, ventral; l, lateral. Scale bars **(A–C)**: 50 μm.

To investigate whether the two morphological types of LPO sensilla (hair-shaped sensilla located distally and club-shaped located basally) house sensory neurons having different projection patterns, mass staining was made from two different sites of the peripheral palp. Fluorescent dye was applied to the transverse cut made at the intermedial and basal part of the third segment, respectively (Figure 3). This classic mass staining labeled both the LPO-specific sensory neurons housed inside the pit and non-LPO sensory neurons located on the outer cuticle and tip of the labial palp. Tracing of the LPO sensory neurons from the basal and intermediate part of the palp demonstrated similar projection patterns in the form of stronger fluorescence intensity in the ipsi- than the contra-lateral LPOG (Figures 3A–C). In the preparations stained from the base (*n* = 8), including both sensillum categories, the mean fluorescence strength was significantly higher within the ipsilateral LPOG (*Mdn* = 65.071) than in the contralateral (*Mdn* = 24.76, *z* = 2.52, *p* = 0.012, *r* = 0.89; Figure 3D). (ii) Likewise, the samples stained at the intermediate part (*n* = 6), including hair-shaped sensilla only, showed a significantly stronger fluorescence intensity in the ipsilateral LPOG (*Mdn* = 25.34) than in the contralateral (*Mdn* = 8.27, *z* = 2.20, *p* = 0.03, *r* = 0.89; Figure 3D). In addition, the mean intensity in the ipsilateral LPOG in the preparations stained from the base was significantly different from the corresponding fluorescence intensity in the preparations stained from the intermediate level (Mann-Whitney *U* test, *U* = 48, *z* = 3.098, *p* < 0.01, *r* = 0.59; Figure 3D). Likewise, the mean intensity in contralateral LPOG in the preparations stained from the base and the intermediate part of the LPO was significantly different (*U* = 41, *z* = 2.91, *p* < 0.05, *r* = 0.82; Figure 3D).

### Exceptional Staining Pattern in the Antennal Lobe

**FIGURE 4:**
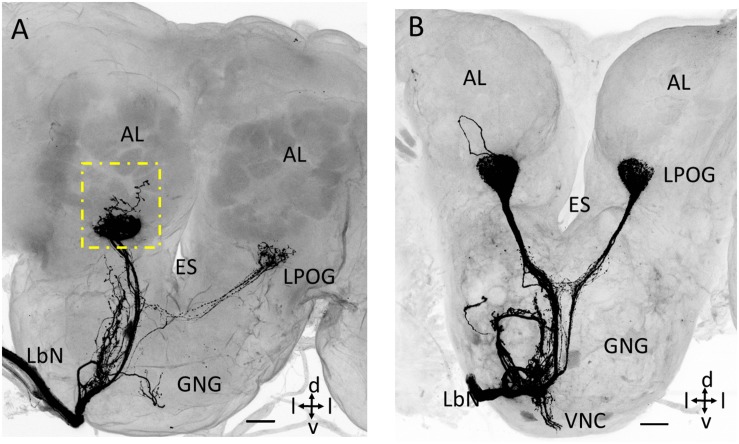
Confocal images showing untypical staining patterns of LPO neurons in the antennal lobe (AL). **(A)** Maximum intensity projection showing, in addition to terminals in the LPO glomerulus (LPOG), a few branches ramifying outside the glomerulus (dotted square box). **(B)** Confocal image showing an axon forming a loop dorsally of LPOG. ES, esophagus; GNG, gnathal ganglion; LbN, Labial nerve; d, dorsal; v, ventral; l, lateral. Scale bars: 50 μm.

Two of the preparations that were stained from the base of the third segment of the labial palp demonstrated antennal-lobe projections having a few branches ramifying outside the LPOG. One preparation showed a short axon projecting to an ordinary glomerulus located postero-medially of the LPOG in the ipsilateral antennal lobe (square box in Figure 4A). The other preparation included a sensory neuron forming an axonal loop dorsally of the LPOG in the ipsilateral antennal lobe, but without targeting any other glomerulus (Figure 4B).

## Discussion

In this study, we traced the central projections of LPO sensory neurons in the moth brain. By performing focused mass staining from the inner pit and the palp surface, respectively, we found that the LPO sensory neurons project to the antennal lobes exclusively. Neurons located on the outer surface of the palp, on the other hand, target the GNG and the ventral nerve cord. Morphological characterization of individual LPO neurons, obtained both by retrograde and anterograde labeling, revealed three sensory neuron types – one ipsilateral, one bilateral, and one contralateral. In addition, mass-staining experiments including selective labeling of hair-shaped and club-shaped LPO sensilla indicated that the sensory neurons housed inside the different sensillum categories innervate the two LPOGs in slightly different manners.

### LPO Sensory Neurons Target the Antennal Lobes Exclusively

Selective staining of sensilla housed inside the pit, carried out for the first time, demonstrated that the LPO sensory neurons of *H. armigera* target the antennal lobes exclusively. This refines the previous findings on central projections of LPO neurons in the current species, reporting about two target areas in addition to the antennal lobes, i.e., the GNG and the ventral nerve cord (Zhao et al., 2013). In the former study, however, a more unspecific staining technique including dye application to the transverse cut surface of the peripheral palp was utilized. Distinct staining of sensilla located on the outer palp surface, as performed here, revealed that the projections originating from this site terminate in the two additional areas – the GNG and the ventral nerve cord. “Merging” the staining patterns obtained from the two labeling techniques utilized in the present study (Figures 1B,C), forms a pattern that is very similar to that previously reported by several researchers using the classic mass staining technique (Figure 1D; Kent et al., 1986; Lee and Altner, 1986; Zhao et al., 2013; Ma et al., 2017). This confirms the suggestion made by Kent et al. (1986) that the axon projections to the LPOGs originated from sensory neurons housed inside the pit and that the branches seen in the GNG were parts of other sensory neuron types. Interestingly, a short peg sensillum type responding to humidity was recently identified on the labial palp of the Asian longhorned beetle, *Anoplophora glabripennis* (Hall et al., 2019).

### Projection Patterns of CO_2_ Sensitive Neurons Across Different Insect Species

The fact that the LPO sensory neurons target the LPOG in each antennal lobe, and no other areas of the central nervous system, means that the projection pattern of CO_2_ sensitive neurons in the moth is quite similar to that found in other insects, including flies and mosquitoes. For example, in the fruit fly, *D. melanogaster*, the CO_2_ sensitive neurons project to a ventrally located antennal-lobe glomerulus, called the V glomerulus (Stocker et al., 1983; Suh et al., 2004), and in the mosquito, *Aedes aegypti*, the corresponding neuron type targets one dorsomedial glomerulus in the antennal lobe (Distler and Boeckh, 1997; Anton et al., 2003; Anton and Rospars, 2004). These two species have ipsilateral projections innervating only one antennal lobe, whereas the moth has a substantial portion of bilateral projections targeting the LPOG in both antennal lobes. Remarkably, yet another well-known mosquito species, *Anopheles gambiae*, has bilateral innervation – like the moth (Anton et al., 2003; Anton and Rospars, 2004).

Notably, there seem to be obvious differences in the peripheral arrangement of the CO_2_ pathway across the various species. Moths have sensilla dedicated to CO_2_ sensory neurons alone (Zhao et al., 2013). Fruit flies and mosquitoes, on the other hand, have their CO_2_ sensitive neurons co-located with olfactory sensory neurons inside olfactory sensilla on the antenna and the maxillary palp, respectively (Riesgo-Escovar et al., 1997; Grant and O’Connell, 2007; Lu et al., 2007).

### Morphology of Individual LPO Neurons

Previous and present mass staining experiments on lepidoptera, including *M. sexta, Pieris rapae*, and *H. armigera*, have demonstrated that both LPOGs receive input from sensory neurons located within each LPO (Kent et al., 1986; Lee and Altner, 1986). Since only a few individual sensory neurons were previously stained (Guerenstein et al., 2004), knowledge about these neurons’ morphologies has been lacking. The three sub-bundles which originate from the labial nerve and project to the LPOGs, as shown in Figures 1A,D, already indicate the three morphological types of LPO sensory neurons identified here: one bilateral, one unilateral, and one contralateral type. To our knowledge, no contralateral CO_2_ sensory neuron has been reported in any other insect order. Since contralateral projections from olfactory or gustatory sensory neurons have never been reported in any insect species, the LPO projections targeting the contralateral LPOG in the moth brain seem to be unique.

### A High Degree of Convergence in the LPOG

Considering the large number LPO sensory neurons, which are housed inside each pit (ca. 1200 sensilla) in *H. armigera* (Zhao et al., 2013), the signal-to-noise ratio in the single target glomerulus is particularly high. The fact that there is a substantial proportion of bilateral projections makes this value even higher. Such an input arrangement might be optimally designed for detecting minor CO_2_ fluctuations (Stange, 1992; Guerenstein et al., 2004). Previous reports have shown that the honey bee and the fruit fly perform simultaneous comparison of the odor concentrations detected by the two antennae in order to trace the source (osmotropotaxis; Martin, 1965; Borst and Heisenberg, 1982; Stocker et al., 1990). Due to the very strong convergence in the CO_2_ system of the moth, we assume a similar mechanism even though the two palps are very closely located.

## Data Availability Statement

All datasets generated for this study are included in the article/Supplementary Material.

## Author Contributions

PKC, XC, PK, and BB contributed to the study concept and design, analysis, and interpretation of data. PKC and XC contributed to the acquisition of data. PKC, XC, PK, X-CZ, and BB contributed to the drafting of the manuscript. PKC, XC, PK, X-CZ, G-RW, and BB contributed to the final manuscript. G-RW and BB contributed to obtaining the funding.

## Conflict of Interest

The authors declare that the research was conducted in the absence of any commercial or financial relationships that could be construed as a potential conflict of interest.

## Footnotes

1 http://imagej.net

## References

[B1] Anton S. Rospars J. P. (2004). Quantitative analysis of olfactory receptor neuron projections in the antennal lobe of the malaria mosquito. *Anopheles gambiae*. *J. Comp. Neurol.* 475 315–326. 10.1002/cne.20174 15221948

[B2] Anton S. Van Loon J. J. A. Meijerink J. Smid H. M. Takken W. Rospars J.-P. (2003). Central projections of olfactory receptor neurons from single antennal and palpal sensilla in mosquitoes. *Arthropod Struct. Dev.* 32 319–327. 10.1016/j.asd.2003.09.002 18089015

[B3] Bogner F. Boppre M. Ernst K. D. Boeckh J. (1986). Co2 sensitive receptors on labial palps of Rhodogastria moths (*Lepidoptera*: *Arctiidae*): physiology, fine structure and central projection. *J. Comp. Physiol. A* 158 741–749. 10.1007/bf01324818 3090241

[B4] Borst A. Heisenberg M. (1982). Osmotropotaxis in *Drosophila melanogaster*. *J. Comp. Physiol.* 147 479–484. 10.1007/bf00612013

[B5] Distler P. Boeckh J. (1997). Central projections of the maxillary and antennal nerves in the mosquito *Aedes aegypti*. *J. Exp. Biol.* 200 1873–1879. 9319784 10.1242/jeb.200.13.1873

[B6] Dong J. Liu H. Tang Q. Liu Y. Zhao X. Wang G. (2014). Morphology, type and distribution of the labial-palp pit organ and its sensilla in the oriental armyworm. *Mythimna separata* (*Lepidoptera*: *Noctuidae*). *Acta Entomol. Sin.* 57 681–687.

[B7] Goyret J. Markwell P. M. Raguso R. A. (2008). Context- and scale-dependent effects of floral Co*_2_* on nectar foraging by *Manduca sexta*. *Proc. Natl.Acad. Sci. U.S.A.* 105 4565–4570. 10.1073/pnas.0708629105 18212123 PMC2290757

[B8] Grant A. J. O’Connell R. J. (2007). “Electrophysiological Responses from Receptor Neurons in Mosquito Maxillary Palp Sensilla,” in *Ciba Foundation Symposium 200 − Olfaction in Mosquito−Host Interactions*, eds Bock G. R. Cardew G. (Hoboken, NJ: Wiley). 10.1002/9780470514948.ch17 8894301

[B9] Guerenstein P. G. Christensen T. A. Hildebrand J. G. (2004). Sensory processing of ambient Co2 information in the brain of the moth *Manduca sexta*. *J. Comp. Physiol. A Neuroethol. Sens. Neural. Behav. Physiol.* 190 707–725. 15235811 10.1007/s00359-004-0529-0

[B10] Hall L. P. Graves F. Myrick A. Hoover K. Baker T. C. (2019). Labial and maxillary palp recordings of the Asian longhorned beetle, *Anoplophora glabripennis*, reveal olfactory and hygroreceptive capabilities. *J. Insect Physiol.* 117 103905. 10.1016/j.jinsphys.2019.103905 31238054

[B11] Homberg U. Christensen T. A. Hildebrand J. G. (1989). Structure and function of the deutocerebrum in insects. *Annu. Rev. Entomol.* 34 477–501. 10.1146/annurev.en.34.010189.002401 2648971

[B12] Kent K. S. Harrow I. D. Quartararo P. Hildebrand J. G. (1986). An accessory olfactory pathway in Lepidoptera: the labial pit organ and its central projections in Manduca sexta and certain other sphinx moths and silk moths. *Cell Tissue Res.* 245 237–245. 3742559 10.1007/BF00213927

[B13] Lee J.-K. Altner H. (1986). Primary sensory projections of the labial palp-pit organ of pieris rapae l. *(Lepidoptera* : *Pieridae)*. *Int. J. Insect Morphol. Embryol.* 15 439–448. 10.1016/0020-7322(86)90036-x

[B14] Lee J.-K. Selzer R. Altner H. (1985). Lamellated outer dendritic segments of a chemoreceptor within wall-pore sensilla in the labial palp-pit organ of the butterfly. *Pieris rapae* L. (*Insecta*, *Lepidoptera*). *Cell Tissue Res.* 240 333–342.

[B15] Lu T. Qiu Y. T. Wang G. Kwon J. Y. Rutzler M. Kwon H. W. (2007). Odor coding in the maxillary palp of the malaria vector mosquito *Anopheles gambiae*. *Curr Biol.* 17 1533–1544. 10.1016/j.cub.2007.07.062 17764944 PMC3113458

[B16] Ma B. W. Zhao X. C. Berg B. G. Xie G. Y. Tang Q. B. Wang G. R. (2017). Central projections of antennal and labial palp sensory neurons in the migratory armyworm mythimna separata. *Front. Cell Neurosci.* 11:370. 10.3389/fncel.2017.00370 29209176 PMC5702295

[B17] Martin H. (1965). Osmotropotaxis in the Honey-Bee. *Nature* 208 59–63. 10.1038/208059a0

[B18] Pallant J. (2007). *Spss Survival Manual*, 3rd Edn New York, NY: McGraw Hill Open University Press.

[B19] Riesgo-Escovar J. R. Piekos W. B. Carlson J. R. (1997). The *Drosophila antenna*: ultrastructural and physiological studies in wild-type and lozenge mutants. *J. Comp. Physiol. A* 180 151–160. 10.1007/s003590050036 9011068

[B20] Sage R. F. (2002). How terrestrial organisms sense, signal, and respond to carbon dioxide. *Integr. Comp. Biol.* 42 469–480. 10.1093/icb/42.3.469 21708741

[B21] Stange G. (1992). High resolution measurement of atmospheric carbon dioxide concentration changes by the labial palp organ of the moth Heliothis armigera (*Lepidoptera*: *Noctuidae*). *J. Comp. Physiol. A* 171 317–324.

[B22] Stange G. (1997). Effects of changes in atmospheric carbon dioxide on the location of hosts by the moth, *Cactoblastis cactorum*. *Oecologia* 110 539–545. 10.1007/s004420050192 28307247

[B23] Stange G. Monro J. Stowe S. Osmond C. B. (1995). The Co2 sense of the moth Cactoblastis cactorum and its probable role in the biological control of the Cam plant Opuntia stricta. *Oecologia* 102 341–352. 10.1007/BF00329801 28306845

[B24] Stocker R. F. Lienhard M. C. Borst A. Fischbach K. F. (1990). Neuronal architecture of the antennal lobe in Drosophila melanogaster. *Cell Tissue Res.* 262 9–34. 10.1007/bf00327741 2124174

[B25] Stocker R. F. Singh R. N. Schorderet M. Siddiqi O. (1983). Projection patterns of different types of antennal sensilla in the antennal glomeruli of *Drosophila melanogaster*. *Cell Tissue Res.* 232 237–248. 10.1007/bf00213783 6411344

[B26] Suh G. S. Wong A. M. Hergarden A. C. Wang J. W. Simon A. F. Benzer S. (2004). A single population of olfactory sensory neurons mediates an innate avoidance behaviour in Drosophila. *Nature* 431 854–859. 10.1038/nature02980 15372051

[B27] Thom C. Guerenstein P. G. Mechaber W. L. Hildebrand J. G. (2004). Floral Co2 Reveals Flower Profitability to Moths. *J. Chem. Ecol.* 30 1285–1288. 10.1023/b:joec.0000030298.77377.7d 15303329

[B28] Zhao X. C. Tang Q. B. Berg B. G. Liu Y. Wang Y. R. Yan F. M. (2013). Fine structure and primary sensory projections of sensilla located in the labial-palp pit organ of Helicoverpa armigera (Insecta). *Cell Tissue Res.* 353 399–408. 10.1007/s00441-013-1657-z 23736380

