# Supplementary material for: Revisiting the Labial Pit Organ Pathway in the Noctuid Moth, Helicoverpa armigera
Source: Front Physiol. 2020 Mar 17;11:202. doi: 10.3389/fphys.2020.00202 (PMC7090773; doi:10.3389/fphys.2020.00202)
Supplement: Supplementary file 1 [file Presentation_1.pdf]

Supplementary Material

1 Supplementary Figures

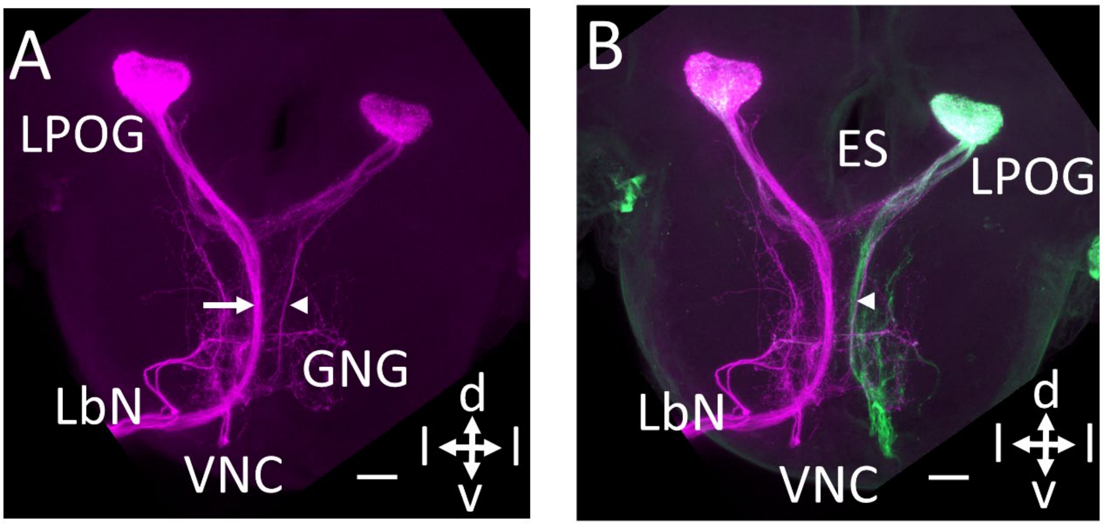

Fig. S1 Double-labeled preparation showing the staining pattern of sensory neurons originating from the terminal segment of the right (*magenta*) and left (*green*) labial palp, respectively. **A**: Confocal image (maximum intensity projection) showing sensory axons from the right palp, stained by Micro-Ruby. A thick sub-branch projects ipsilaterally (arrow) whereas a thin sub-branch projects contralaterally (arrowhead). **B:** Double-labeling showing an overlay of the Micro-Ruby staining from the right palp (*magenta*) and Alexa-488 staining from the left palp (*green*). The thick fiber bundle from the left palp merges with the thin sub-bundle from the right palp (arrowhead). (The preparation is slightly overexposed.) ES, esophagus; VNC, ventral nerve cord; LbN, labial nerve; d, dorsal; v, ventral; l, lateral. Scale bars: 50 µm.

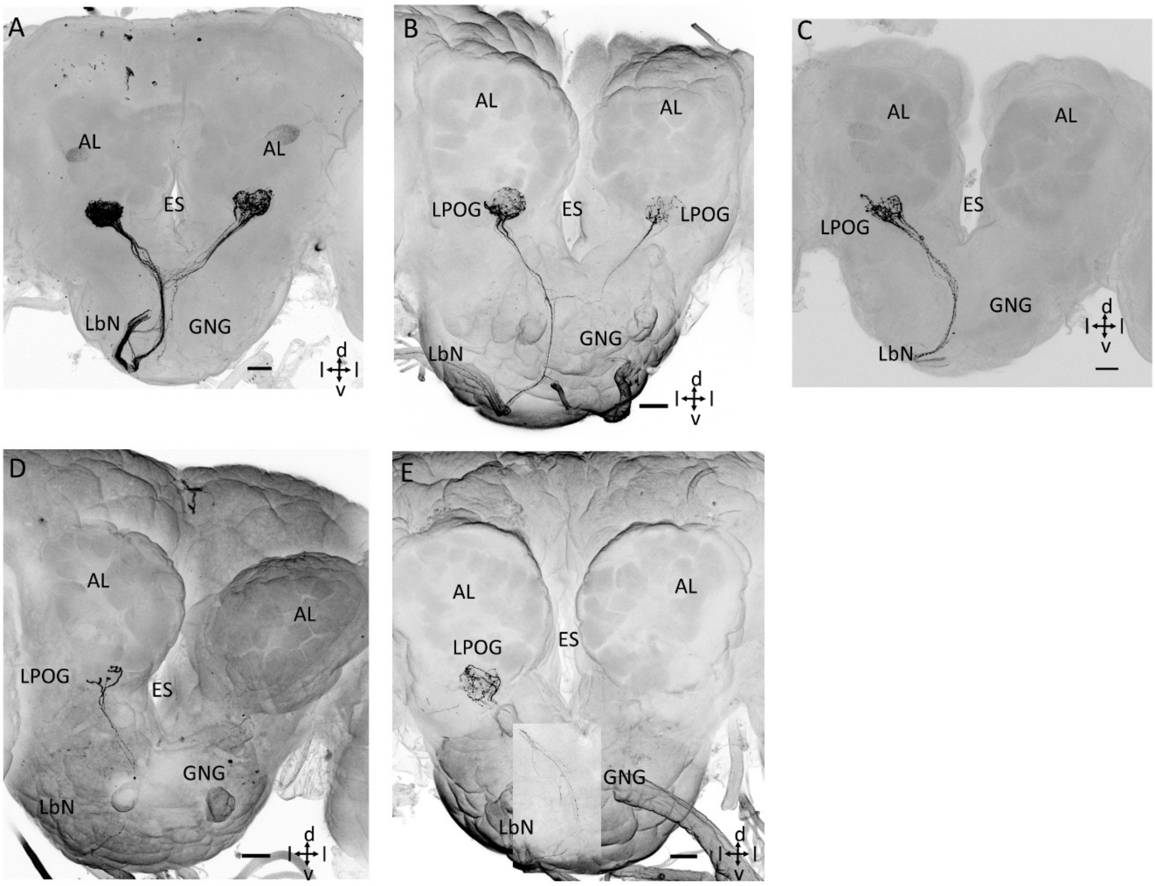

Fig. S2 Confocal images of five preparations, labelled via focused mass staining from the labial pit organ (LPO). **A-E**: All preparations showed a principally similar staining pattern including terminal branches in the LPO glomerulus (LPOG) exclusively. The labeling in the ipsilateral LPOG was generally stronger than in the contralateral LPOG. AL, antennal lobe; ES, esophagus; GNG, gnathal ganglion; LbN, labial nerve; d, dorsal; v, ventral; l, lateral. Scale bars: 50 µm.

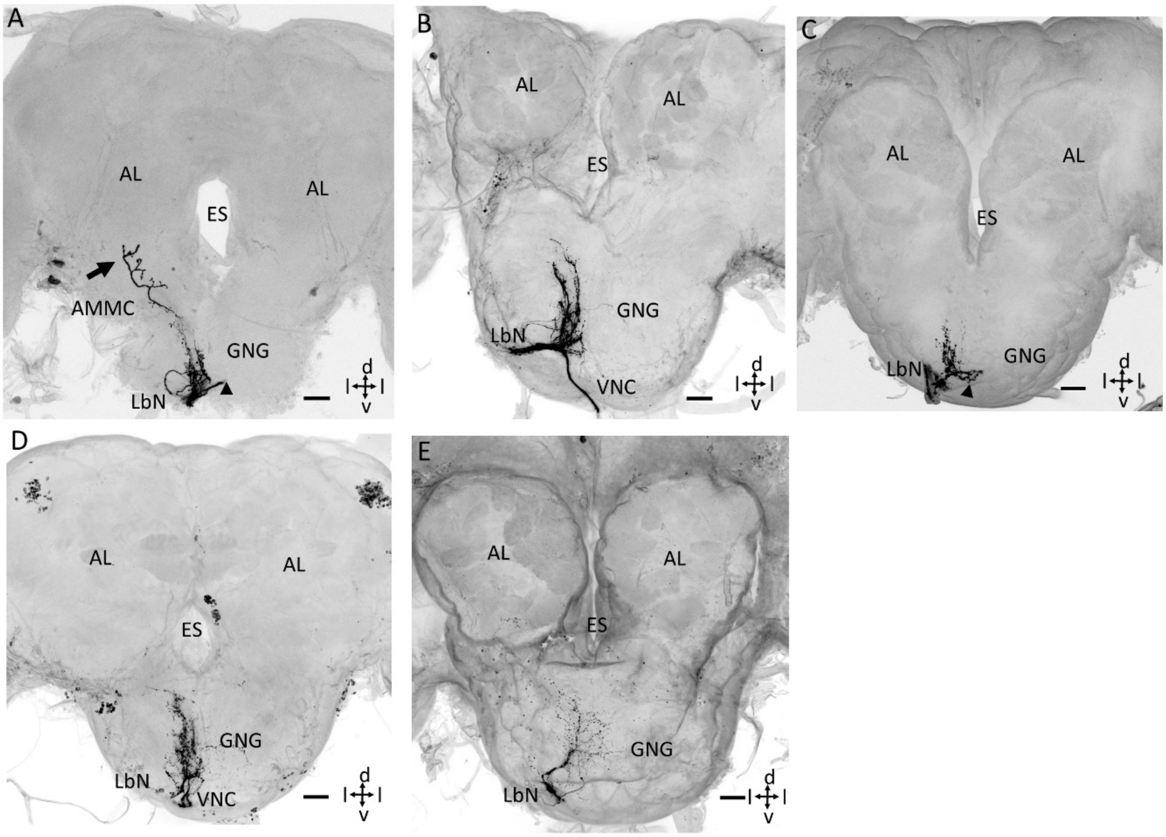

Fig. S3 Confocal images of five preparations, labelled via mass staining from the outer cuticle of the labial palp (longitudinal section). **A-E**: All preparations showed a principally similar staining pattern including terminal branches in the gnathal ganglion (GNG), the antennal mechanosensory and motor center (AMMC), and the ventral nerve cord (VNC). Generally, no labeling was visualized in the antennal lobe (AL). ES, esophagus; LbN, labial nerve; d, dorsal; v, ventral; l, lateral. Scale bars: 50 μm.

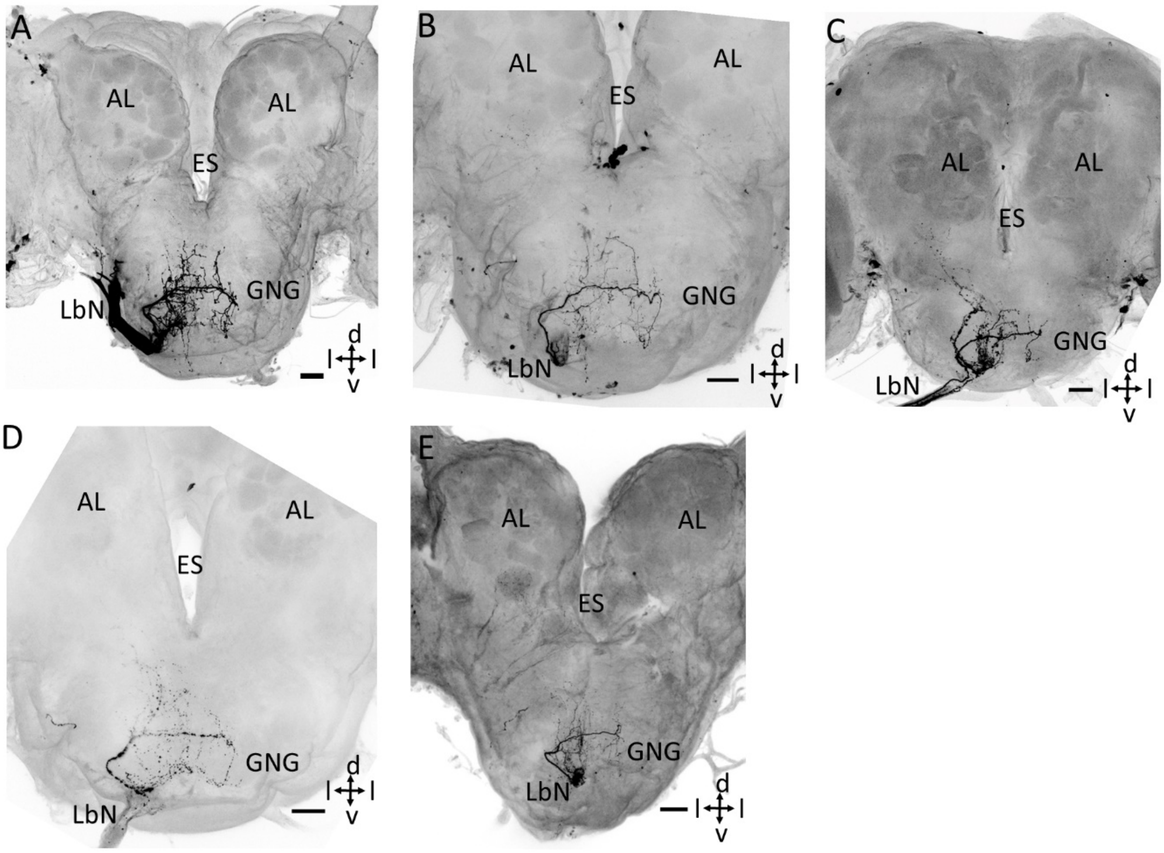

Fig. S4 Confocal images of five preparations, labelled via mass staining from the outer tip of the labial palp. **A-E**: All preparations showed a principally similar staining pattern including bilateral terminal branches in the gnathal ganglion (GNG). No labeling was visualized in the antennal lobe (AL). ES, esophagus; LbN, labial nerve; d, dorsal; v, ventral; l, lateral. Scale bars: 50 µm.
